# Plasma miR-19b and miR-183 as Potential Biomarkers of Lung Cancer

**DOI:** 10.1371/journal.pone.0165261

**Published:** 2016-10-21

**Authors:** Ivan A. Zaporozhchenko, Evgeny S. Morozkin, Tatyana E. Skvortsova, Anastasia A. Ponomaryova, Elena Yu Rykova, Nadezhda V. Cherdyntseva, Evgeny S. Polovnikov, Oksana A. Pashkovskaya, Evgeny A. Pokushalov, Valentin V. Vlassov, Pavel P. Laktionov

**Affiliations:** 1 Institute of Chemical Biology and Fundamental Medicine of SB RAS, Novosibirsk, Russia; 2 Novosibirsk Research Institute of Circulation Pathology of Academician E.N. Meshalkin, Novosibirsk, Russia; 3 Tomsk Cancer Research Institute of SB RAMS, Tomsk, Russia; 4 National Research Tomsk Polytechnic University, Tomsk, Russia; 5 National Research Tomsk State University, Tomsk, Russia; University of California San Francisco, UNITED STATES

## Abstract

Lung cancer is a complex disease that often manifests at the point when treatment is not effective. Introduction of blood-based complementary diagnostics using molecular markers may enhance early detection of this disease and help reduce the burden of lung cancer. Here we evaluated the diagnostic potential of seven plasma miRNA biomarkers (miR-21, -19b, -126, -25, -205, -183, -125b) by quantitative reverse transcription PCR. Influence clinical and demographical characteristics, including age, tumor stage and cancer subtype on miRNA levels was investigated. Four miRNAs were significantly dysregulated (miR-19b, -21, -25, -183) in lung cancer patients. Combination of miR-19b and miR-183 provided detection of lung cancer with 94.7% sensitivity and 95.2% specificity (AUC = 0.990). Thus, miRNAs have shown the potential to discriminate histological subtypes of lung cancer and reliably distinguish lung cancer patients from healthy individuals.

## Introduction

Lung cancer (LC) is currently the world’s leading cause of cancer-related mortality with an overall mortality to incidence ratio of 0.87 [1]. Only 15% of lung cancer patients are diagnosed at an early stage of the disease, whereas 56% of the patients have already developed distant metastases at the moment of cancer detection. The former group has 5-year survival rate of 54%, while for the latter it is as low as 4% [2]. This is mainly due to late manifestation of symptoms and high degree of tumor heterogeneity within the established histological types. As a result, most patients are only diagnosed at the advanced stages of the disease, which greatly decreases the chances of a positive outcome. Additionally since lung cancer is a diverse group of malignancies, the effect of therapeutic measures can also differ dramatically on a case-to-case basis [3].

Current methods used to diagnose lung cancer include chest radiography, computed tomography (CT), magnetic resonance imaging (MRI) and histopathology of resected tumor tissue samples, bronchoscopy biopsies, fine needle aspirations or sputum analysis. Instrumental methods can be followed up by molecular diagnostics to identify relevant genetic alterations, such as EGFR mutations and ALK fusions [4]. However, the system is less than ideal. Chest radiography commonly used for screening involves health risks and only reliably detects nodules of 0.5–1 cm, corresponding to an advanced stage of the disease [5]. Unfortunately, most other methods can’t be used for routine screening because of high costs (CT), invasiveness (biopsy) or low prevalence of marker in population. Although low dose CT was shown to be a promising screening tool, its implementation into general practice is hurdled by high false positive rates (overdiagnosis), uncertain costs-benefits profile and unexplored health risks [6]. MRI is a costly method with low processivity and high false positive rates that is mostly used to confirm an existing diagnosis and search for metastases. New molecular methods (e.g. Epi proLung by Epigenomics AG) are yet to be approved for clinical use and often use inconvenient (lavage) or invasive (tumor tissue) sources of diagnostic material. Thus, the problem of early lung cancer detection persists and new strategies for preclinical screening and monitoring of post therapy relapses are required to relieve the burden of lung cancer.

A possible solution may come in form of complementary diagnostics using blood-based molecular markers. One class of prospective biomarkers is miRNA–short, non-coding regulators of gene expression that are relatively stable in circulation [7]. Aberrant miRNA expression is a feature of most human cancers and can either drive the malignant transformation or result from dysregulation caused by cancer [8]. Specific subsets of miRNAs that reflect important properties of the tumor were found circulating in blood of cancer patients [7]. These signatures can serve as potential biomarkers for cancer diagnosis and theranostics. To date a handful of candidate biomarker miRNAs have been identified for further investigation with at least two panels of prospective miRNA markers going through clinical trials [9,10]. In this study, we evaluated seven promising miRNA biomarkers previously reported to be dysregulated in lung cancer and involved in regulation of cell cycle and apoptosis, tumor development, invasion and vascularization. Using recently developed method for miRNA isolation [11] and quantitative reverse transcription PCR (qRT-PCR) we have compared relative expression of seven miRNAs (miR-21, -19b, -25, -183, -125b, -126, -205) in blood plasma of 50 healthy individuals and 75 lung cancer patients.

## Materials & Methods

### Selection of plasma miRNAs

Many reports have demonstrated the diagnostic value of miRNA detection for the detection of lung cancer. The inclusion criteria for this study were that miRNAs should: (1) regulate targets involved in lung cancer development; (2) regulate an array of key oncogenic pathways; (3) be detectable in plasma; (4) be readily analyzed by qRT-PCR and have pronounced differences between health and disease; (5) have additional diagnostic relevance or potential for theranostic application. From the pool of potential biomarkers, seven miRNAs (Table 1) were selected as targets for evaluation.

**Table 1:** Targets and functions of selected miRNAs.

miRNA	Known targets and functions^a^	References
**miR-21**	OncomiR, regulates genes involved in apoptosis (PTEN, BCL2, PDCD4) and angiogenesis.	[12–16]
**miR-19b**	OncomiR, enhances proliferation and angiogenesis by regulation of TP53, BCL2, PTEN and Prkaa-1.	[17,18]
**miR-25**	OncomiR, suppresses the expression of proteins involved in regulation of apoptosis and cell cycle (BCL2L11, CDKN1C).	[19,20]
**miR-183**	OncomiR, regulates signal transduction pathways (EGR1, PTEN), and genes crucial for cell migration, invasion (Ezrin) and glucose metabolism.	[16,21,22]
**miR-125b**	OncomiR or oncosupressor, regulates cell cycle and apoptosis through p53, BAK и erbB2-3.	[23,24]
**miR-126**	Oncosupressor, regulates proto-oncogenes such as KRAS and p38, vascular endothelial growth factor VEGF-A и DNA methyl transferase 1 (DNMT1).	[12,13,16,19]
**miR-205**	Regulates tumor-suppression genes PTEN, PHLPP, ERBB3.	[13,15,16]

^a^According to miRTarBase (http://mirtarbase.mbc.nctu.edu.tw/).

### Study population

Blood samples of 50 healthy individuals were obtained from Center of New Medical Technologies of ICBFM SB RAS (Novosibirsk, Russia) and Novosibirsk Research Institute of Circulation Pathology of Academician E.N. Meshalkin (Novosibirsk, Russia) (Table 2). Samples of 75 lung cancer patients with no previously known history of cancer were obtained from Novosibirsk Research Institute of Circulation Pathology of Academician E.N. Meshalkin (Novosibirsk, Russia) and Cancer Research Institute of RAMS (Tomsk, Russia) (Table 2). Most patients were diagnosed with stage II or stage III lung cancer, no patients with stage I were available due to late diagnosis. Lung biopsy specimens and imaging techniques were applied to confirm histopathological features and tumor stages of lung cancer patients. None of the patients have undergone surgical treatment or received chemotherapy prior or at the time of blood sampling. All blood samples were collected between January 2013 and March 2014.

**Table 2:** Overview of the study population.

Characteristic	Lung cancer patients n = 75	Healthy individuals n = 50
**Age**		
Mean±SD	65,0±9,0	51,2±8,8
Range	(31–79)	(35–64)
**Gender**		
Male	67	42
Female	8	8
**Non-smokers**	7	8
**Tumor stage**		
I	-	
II (2a, 2b)	24	
III	47	
IV	4	
**Tumor subtype**		
Non-small cell lung cancer (NSCLC)	71	
Squamous cell carcinoma (SCC)	53	
Adenocarcinoma (AD)	18	
Small cell lung cancer (SCLC)	4	

All procedures performed in studies involving human participants were in accordance with the ethical standards of the institutional and/or national research committee and with the 1964 Helsinki declaration and its later amendments or comparable ethical standards. Study was approved by ethical committees of ICBFM SB RAS and Novosibirsk Research Institute of Circulation Pathology of Academician E.N. Meshalkin. Full written informed consent was provided by all participants. This article does not contain any studies with animals performed by any of the authors.

### Blood plasma collection and miRNA isolation

Blood plasma samples were prepared as follows. Venous blood was collected in EDTA spray-coated vaсutainers (BD, Cat. No. 368589) and processed within 4 hours after blood sampling. To obtain plasma blood was centrifuged at 400×g for 20 min, supernatant was transferred into a new tube and centrifuged at 800×g for 20 min. Resulting supernatant was stored frozen in aliquots at −80°C.

To isolate RNA frozen plasma samples were thawed and centrifuged for 5 min at 3,000×g to rid of the cryoprecipitate. RNA was isolated from the supernatant using single-phase phenol-free extraction protocol described previously [11]. Briefly, plasma was incubated with single-phase extraction solution and total miRNA was purified on silica-based spin columns (BioSilica Ltd, Novosibirsk, Russia), co-precipitated with glycogen and dissolved in RNase-free water.

### Reverse transcription and quantitative TaqMan PCR

Primers and probes for reverse transcription and TaqMan qPCR listed in S1 Table were synthesized in the Laboratory of Medicinal Chemistry (ICBFM SB RAS, Novosibirsk).

Reverse transcription (RT) of miRNA templates was performed as described previously [25]. Each RT reaction was performed in a total volume of 10 μl and contained 3 μl RNA, 50 nM each miRNA-specific primer, 1 unit RiboLock RNAse inhibitor (Thermo Scientific, Lithuania), 100 units MMLV reverse transcriptase (Thermo Scientific, Lithuania), 2μl 5× MMLV buffer (250 mM Tris-HCl (pH 8.3 at 25°C), 250 mM KCl, 20 mM MgCl_2_, 50 mM DTT) (Thermo Scientific, Lithuania), and 250 μM each dNTP. The reaction conditions were as follows: 16°C– 30 min, 42°C– 30 min, 70°C– 10 min. Samples without RNA template and preparations of genomic DNA were used as negative controls.

Each qPCR reaction contained 2.5 μl RT product, 1.25 unit Taq DNA polymerase (BiolabMix, Russia), 3 μl 10× PCR buffer (750 mM Tris-HCl (pH 8.8 at 25°C), 200 mM (NH_4_)_2_SO_4_, 0.1% (v/v) Tween 20), 4 mM MgCl_2_, 250 μM each dNTP, 600 nM forward primer, 800 nM universal reverse primer, and 300 nM miRNA-specific TaqMan probe in a total volume of 30 μl. All reactions were performed in duplicates. Real-time PCR amplification was run on the iCycler iQ5 Real-Time PCR Detection System (Bio-Rad, USA). After initial denaturation at 95°C for 3 min, the reactions were run for 50 cycles at 95°C for 15 s and 60°C for 45 s.

### Survival analysis

Patient survival outcomes were analyzed using the Kaplan-Meier cumulative proportion survival curves to assess the prognostic value of miRNAs expression in lung cancer. Expression of each miRNA in sample was compared to the group median to form the “High” and “Low” miRNA expression groups. Patients were followed for up to 25 months after blood sampling. Cases with incomplete records, non-uniform treatments and cancer-unrelated mortality were excluded from the analysis. Kaplan-Meier survival analysis and Logrank tests were performed using MedCalc software.

### Data analysis

Quantification cycle values (Cq) for all miRNAs yielded by qRT-PCR were normalized to miR-16 which is an abundant miRNA often used as endogenous control [26]. Normalizing was done by subtracting Cq values for miR-16 from Cq values for target miRNA for each sample. No significant differences in miR-16 expression levels were observed between lung cancer patients and healthy individuals (P = 0.2910) or between lung cancer patients with SCC or AD (P = 0.8775) (S2 Table).

Delta Cq (dCq) values were further analyzed using MedCalc software. Pairwise comparisons on normally distributed data were performed using independent samples T-tests (equal variances) or Welch-test (unequal variances). One- or Two-way ANOVA was used for comparisons involving more than two groups. To assess the diagnostic performance of miRNAs Receiver Operating Characteristic (ROC) curves were used. Optimal cut-off criterion value was determined by Youden’s index. All tests were considered statistically significant at P<0.05 unless specified otherwise.

## Results

**Fig 1:**
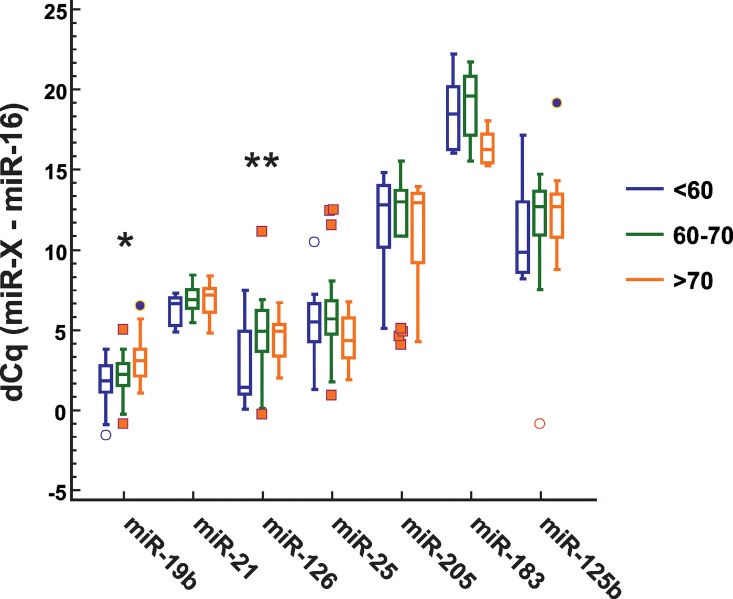
MiRNA expression in age groups. Box plots of relative miRNA expression levels in plasma of lung cancer patients divided into three age groups: <60 years, 60–70 years, >70 years. The expression levels of miRNAs were normalized to miR-16 using dCq method. *P = 0.004; **P = 0.007 (One-way ANOVA).

First, dependence of plasma miRNA expression levels from demographic characteristics of lung cancer patients was investigated. No direct correlations between age or gender and levels of any of the tested miRNAs were found.However, when participants were divided into three age groups (0–59, 60–69, 70+ years) significant differences in expression levels of miR-19b and miR-126 in <60 years and 60–69 years and >70 years groups have been discovered (Fig 1).

To identify the connections between clinico-pathological charateristics and expression levels of miRNAs, we investigated the relationships between stage and subtype of lung cancer and plasma expression levels of miRNAs using Two-way ANOVA. No interrelation between both stage and subtype has been found for any of the miRNAs. For miR-205 statistically significant (P = 0.022) connection between subtype and plasma level was discovered, miR-19b showed a similar trend, that was not strictly significant (P = 0.069) (S1 Fig). Additionally, miR-126 exhibited an even weaker trend of relation between stage and plasma level (P = 0.083).

**Fig 2:**
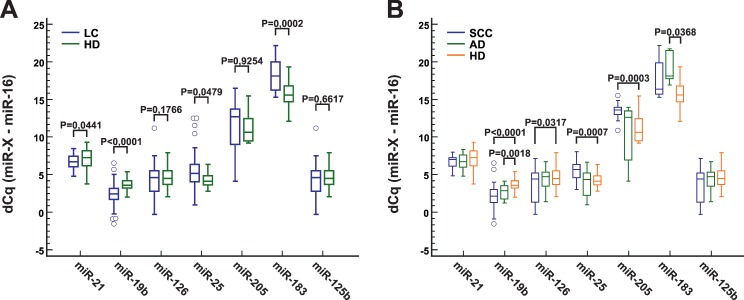
MiRNA expression in plasma. Box plots of relative miRNA expression levels in plasma of lung cancer patients and healthy individuals. The expression levels of miRNAs were normalized to miR-16 using dCq method. (А) Lung cancer patients (LC) and healthy individuals (HD); (B) Squamous cell carcinoma (SCC), adenocarcinoma (AD) patients and healthy individuals (HD). P-values obtained by T-test (two-sided) or Welch-test (two-sided).

Expression levels of four miRNAs (miR-19b, -21, -25, -183) were significantly different (p<0.05, T-test, two-sided) between lung cancer patients and healthy individuals (Fig 2A). Two of the miRNAs were up-regulated (miR-19b and miR-21), two were down-regulated (miR-25 and miR-183) in cancer patients, but difference in dCq values between cancer and health groups was only substantial for three miRNAs–miR-19b (1.25), miR-25 (-1.09) and miR-183 (-2.49) (S3 Table). Four miRNAs (miR-19b, -126, -25, -205) were differently regulated in SCC patients when compared to healthy controls (Fig 2B). In AD patients only miR-19b and miR-183 were differently expressed and miR-25 was nearly statistically differently expressed (Fig 2B). Only miR-19b was universally dysregulated across lung cancer group, regardless of subtype, while in general the profiles of miRNA expression in plasma were different between lung cancer subtypes. In contrast to the majority of published data, in our study miR-183 was down-regulated rather than up-regulated in lung cancer patients. One explanation is that we measured the 5p strand and not 3p strand of miR-183 (this issue is examined in more detail in the Discussion section). Interestingly, the difference of miR-21 levels between lung cancer patients at large and healthy individuals was only 1.36-fold but still statistically significant (P = 0.0441), but no statistical differences in miR-21 expression were found for either SCC or AD alone.

**Fig 3:**
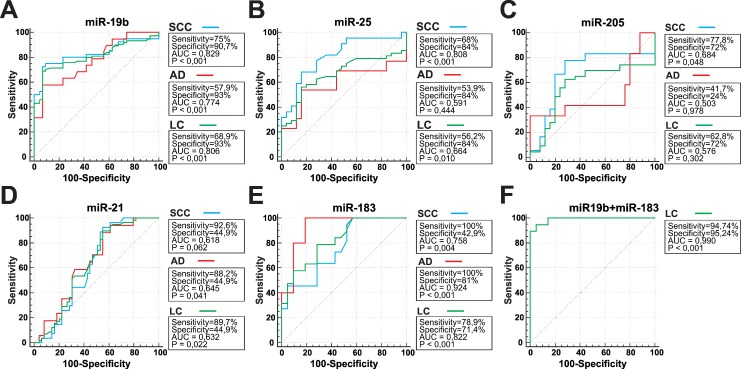
ROC analysis of miRNA expression. Receiving Operator Characteristic (ROC) curves for individual miRNAs and combination of miR-19b and miR-183. (A)–(E) Individual miRNAs; (F) Binary logistic regression of miR-19b and miR-183. ROC curves discriminate squamous cell carcinoma (SCC), adenocarcinoma (AD) and total study population of lung cancer patients (LC) from healthy individuals (HD).

To test whether different expression profiles result in different diagnostic performance ROC analysis of differentially expressed miRNAs was performed (Fig 3). Among the tested miRNAs, miR-19b showed the highest diagnostic value for total study population of LC patients and SCC, while miR-183 was more effective in discriminating AD from healthy individuals. On this basis, we presumed that combination of miRNAs with opposite bias should provide a more potent diagnostic tool for cancer detection in general population than individual miRNAs or panels of miRNAs that are specific to just one lung cancer subtype. Indeed, stepwise binary logistic regression identified the combination of miR-19b and miR-183 as a strong predictor of lung cancer and yielded a noticeable increase in AUC: 0.990 (miR-19b+miR-183) versus 0.806 (miR-19b) and 0.924 (miR-183). This criterion can be used to identify lung cancer with 94.7% sensitivity and 95.2% specificity thus surpassing the use of any of the tested miRNA individually.

**Fig 4:**
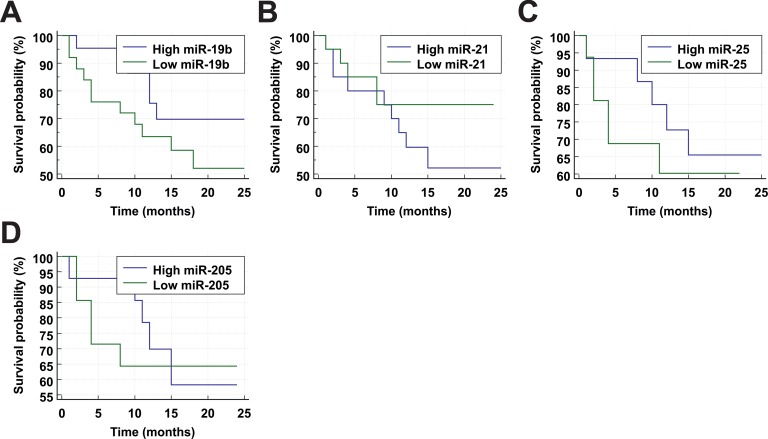
Survival analysis of LC patients. Kaplan-Meier survival analysis of lung cancer patients. (A)–(D) Expression of miRNAs and survival of lung cancer patients. The ‘High’ and ‘Low’ miRNA expression groups were delimited by group median dCq value for each miRNA.

Since miRNAs are often linked to prognosis and outcomes we performed survival analysis to see if there is a direct link between patient survival and plasma expression levels of the tested miRNAs (Fig 4). Patients were followed for up to 25 months after blood sampling. Cases with incomplete records, non-uniform treatments and cancer-unrelated mortality were excluded from the analysis. The ‘High’ and ‘Low’ miRNA expression groups were delimited by group median dCq value for each miRNA.

Patients with high plasma expression levels of miR-19b demonstrated longer survival times than those with low expression of this miRNA. This effect (as well as overall cancer related mortality) was less pronounced in older age (S2 Fig). Low expression level of miR-21 had a positive effect on survival after 10 months mark, which is consistent with many previous studies [27]. In contrast, low expression levels of miR-205 and miR-25 can be indicators of reduced short term (<10 months) mortality. No noticeable trends in patients’ survival were identified for miR-183 (S3 Fig). Unfortunately, since Logrank test has failed to identify any significant differences in survival curves between high and low expression groups, described trends would require further confirmation on larger sample pools complete with follow-up data.

## Discussion

Here, we describe differences in relative expression levels of miRNAs circulating in plasma of patients with different subtypes of lung cancer using a panel of prospective miRNA markers. Even more importantly, implications of these differences on the overall performance of miRNAs as potential biomarkers for lung cancer screening are illustrated. In this study, we chose to focus on two major subtypes of lung cancer–SCC and adenocarcinoma AD. In the US, according to National Cancer Institute, SCC and AD account for 25% and 40% of lung cancer cases respectively. However, several studies have demonstrated that in Europe SCC is still the leading lung cancer type, although AD incidence is increasing [28]. According to Russian Cancer Research Center, similar distribution and tendencies of lung cancer incidence are observed in Russian population. In the cohort used in this study, approximately 94% of patients were diagnosed with either SCC or AD.

Within the limits of the panel, we have identified distinct signatures for both subtypes–miR-19b, miR-126, miR-25, and miR-205 for SCC and miR-19b and miR-183 for AD. The resulting miRNA signature for lung cancer in general was miR-21, miR-19b, miR-25 and miR-183. A combination of two miRNAs–miR-19b and miR-183 has been identified as strong predictor of lung cancer.

The miR-19b is a part of human oncogene miR-17-92 cluster and was reported to be involved with development of several human cancers [29]. It was previously shown to be dysregulated in lung cancer, which contributes to cancer development by inhibiting apoptosis through PTEN and TP53 [30, 31]. This miRNA has been previously identified as a marker of NSCLC and SCC in particular. For example in Boeri et al [32] ratios based on miR-19b up-regulation were included in the signature of risk and signature of diagnosis. Both our data and previous research demonstrate that high expression of miR-19b is a characteristic of disease [32, 17, 18]. One study reported an opposite effect of miR-19b expression on survival of lung cancer patients. Wu et al [18] demonstrated that tissue expression of miR-19b is associated with higher TNM stage, lymph node metastasis and poorer survival and its serum concentration correlated with poor prognosis. Previous reports [17] also show miR-19b to be a specific marker of SCC, which supports our current findings.

Changes in the expression of miR-183 are also associated with many cancers including lung cancer, breast cancer, prostate cancer, colorectal cancer, osteosarcoma and others [21, 16]. This miRNA is involved in regulation of migration and invasion by targeting Ezrin [33] and also controls key signal pathways through PTEN and EGR1 [34]. In addition to its primary connection to metastasing, it was also shown to have the potential to differentiate between SCC and AD [34]. In contrast to the majority of published data, in our study, miR-183 was down-regulated rather than up-regulated in lung cancer patients. These data could be concerned with the differences in miRNA isolation protocols or effects of different study populations. However, many studies involving miR-183 do not expressly state which particular strand of mir-183 – 3p or 5p—was investigated. In this study, we measured the expression of miR-183-5p, which is also known as miR-183* and was previously shown to be down-regulated in LC [35]. Along with previous reports of up-/down-regulation of miR-183 species, our data hints at the possibility of miR-183/miR-183* ratio being altered in LC.

Surprisingly, neither miR-205 nor miR-25 both previously reported to differentiate between SCC and AD [36, 37, 38] directly discriminated between the subtypes in any of the performed assays. However, they have both demonstrated a tendency to discriminate SCC rather than AD from healthy controls, although with low diagnostic significance (Fig 3).

Development of miRNA biomarkers for clinical use currently faces several major problems aptly summarized by Sozzi et al [39]. Individual miRNAs, although experimentally being strong predictors of disease and outcomes may not be suitable for use in clinical practice due to small differences in expression levels and high individual variance resulting into an overlap between healthy and diseased subjects. This pitfall can be avoided by using a panel of several miRNAs. In most cases, miRNA panels are put together based on statistical findings using high throughput methods. However, we argue that to be useful for making clinical decisions composition of the panel should be initially set to reflect tumor genetics and phenotype, and account for both inter- and intra-tumor heterogeneity. Our study confirms this thesis, although the differences in lung cancer subtypes between populations must be further investigated.

Another known problem is setting the standards for methodology employed to measure the expression of endogenous miRNAs in biological fluids, particularly serious issue being the lack of proper internal references. Previous studies have identified severe limitations of many individual reference RNAs such as U6 and miR-16, including high sensitivity to sample quality (haemolysis), handling and storage conditions [40]. This may explain the less than impressive performance of some of the markers in this study and can account for the lack of reproducibility of many previous miRNA biomarker findings. A more reasonable option than using a universal reference is to select a reference miRNA (or a panel of miRNAs) individually for each biomarker panel, which requires additional extensive verification steps. Another alternative is to establish functional miRNA pairs and construct miRNA ratios, effectively having individual references for every miRNA biomarker in the panel as described for example in Boeri et al [32]. This approach also allows to integrate the biology of miRNAs with empirical data of their expression through knowledge of their targets and effects.

## Conclusion

The performance of seven promising miRNA markers was evaluated using qRT-PCR. Expression levels of four selected miRNAs were shown to be different in lung cancer patients as compared to healthy individuals. Combination of miR-19b and miR-183 was shown to predict lung cancer with 94.7% sensitivity and 95.2% specificity. At the same time, a more profound analysis is required to establish new biomarkers for efficient detection and post-treatment follow-up of lung cancer patients.

## Supporting Information

S1 Fig Dependence of miRNA expression on stage and subtype of LC. Box plots of miRNA expression levels in different lung cancer subtype and stage of disease. Squamous cell carcinoma (SCC) and adenocarcinoma (AD) patients vs healthy individuals (HD).
(JPG)

S2 Fig Kaplan-Meier survival analysis of lung cancer patients. Expression levels of miR-19b and survival of lung cancer patients across the age groups: <60 years (A), 60–70 years (B), >70 years (B). ‘Low’ and ‘High’ expression groups are based on the expression levels of miR-19b in plasma of lung cancer patient in relation to the group median dCq value for the respective miRNA.
(JPG)

S3 Fig Kaplan-Meier survival analysis of lung cancer patients. Expression levels of miR-183 and survival of lung cancer patients. ‘Low’ and ‘High’ expression groups are based on the expression levels of miR-183 in plasma of lung cancer patient in relation to the group median dCq value.
(JPG)

S1 Table Sequences of primers and probes used for reverse transcription and TaqMan qPCR. (DOCX)

S2 Table Stability of miR-16 expression in the study population. (DOCX)

S3 Table Fold-change and significance levels of miRNA expression levels between groups. (DOCX)
