# Supplementary figures and images for: Plasma miR-19b and miR-183 as Potential Biomarkers of Lung Cancer
Source: PLoS One. 2016 Oct 21;11(10):e0165261. doi: 10.1371/journal.pone.0165261 (PMC5074500; doi:10.1371/journal.pone.0165261)

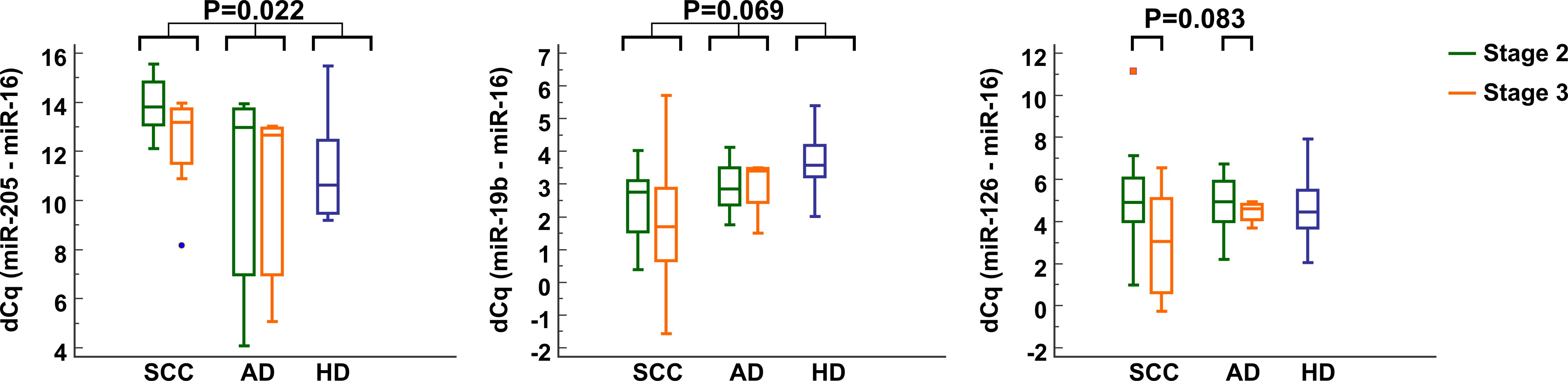

Supplement: S1 Fig — Box plots of miRNA expression levels in different lung cancer subtype and stage of disease. Squamous cell carcinoma (SCC) and adenocarcinoma (AD) patients vs healthy individuals (HD). (JPG) [file pone.0165261.s001.jpg]

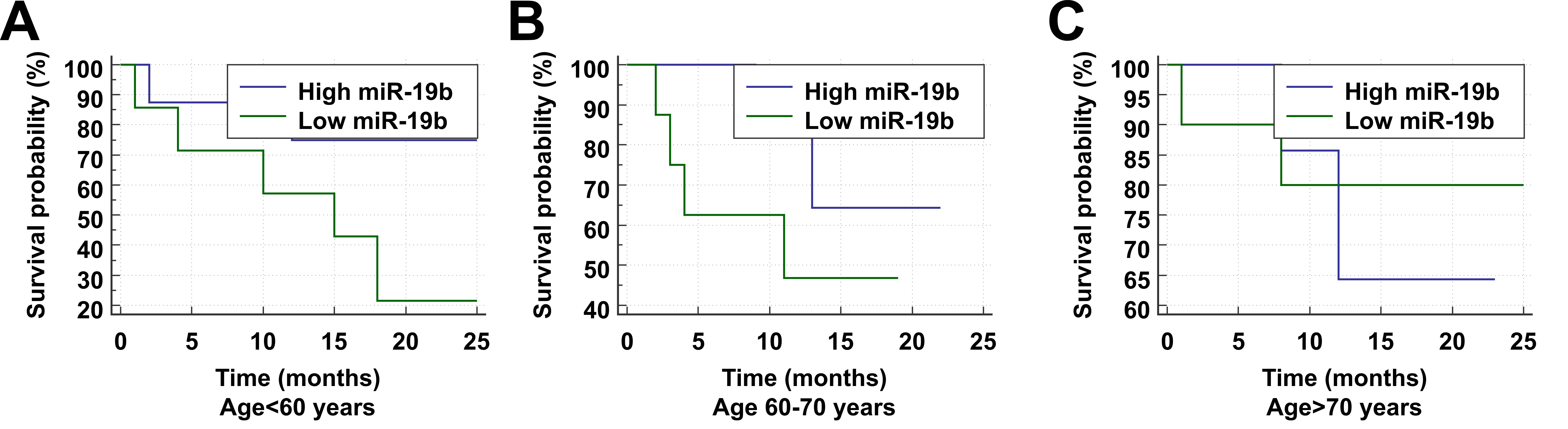

Supplement: S2 Fig — Expression levels of miR-19b and survival of lung cancer patients across the age groups: <60 years (A), 60–70 years (B), >70 years (B). ‘Low’ and ‘High’ expression groups are based on the expression levels of miR-19b in plasma of lung cancer patient in relation to the group median dCq value for the respective miRNA. (JPG) [file pone.0165261.s002.jpg]

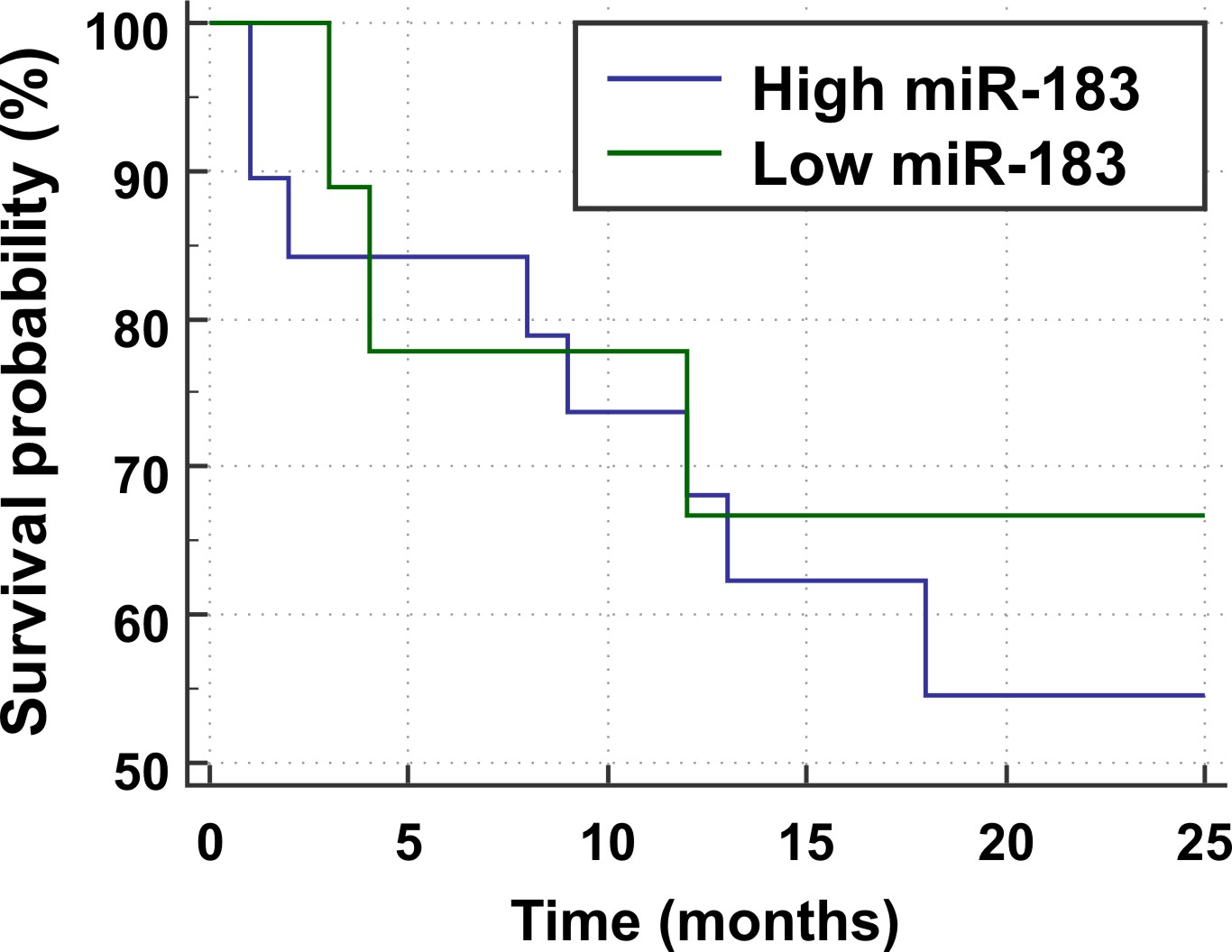

Supplement: S3 Fig — Expression levels of miR-183 and survival of lung cancer patients. ‘Low’ and ‘High’ expression groups are based on the expression levels of miR-183 in plasma of lung cancer patient in relation to the group median dCq value. (JPG) [file pone.0165261.s003.jpg]
