# Supplementary material for: Plasma miR-19b and miR-183 as Potential Biomarkers of Lung Cancer
Source: PLoS One. 2016 Oct 21;11(10):e0165261. doi: 10.1371/journal.pone.0165261 (PMC5074500; doi:10.1371/journal.pone.0165261)
Supplement: S1 Table — (DOCX) [file pone.0165261.s004.docx]

**Table S1. Sequences of primers and probes used for reverse transcription and TaqMan qPCR.**

| **Universal Reverse Primer** | 5’-GTGCAGGGTCCGAGGT-3’ |
| --- | --- |
| **hsa-miR-16-5p**  **(miR-16)** | RT: 5’-GTCGTATCCAGTGCAGGGTCCGAGGTATTCGCACTGGATACGACCGCCAA-3’  Forward: 5’-GCCCGTAGCAGCACGTAAATAT-3’  Probe: 5’-(FAM)-GCACTGGATACGACCGCCAA-(FQ)-3’ |
| **hsa-miR-21-5p**  **(miR-21)** | RT: 5’-GTCGTATCCAGTGCAGGGTCCGAGGTATTCGCACTGGATACGACTCAACA-3’  Forward: 5’-GCCCGCTAGCTTATCAGACTGAT-3’  Probe: 5’-(FAM)-GCACTGGATACGACTCAACA-(FQ)-3’ |
| **hsa-miR-19b-3p**  **(miR-19b)** | RT 5’-GTCGTATCCAGTGCAGGGTCCGAGGTATTCGCACTGGATACGACTCAGTT-3’  Forward: 5’-CGCTGTGCAAATCCATGCAA-3’  Probe: 5’-(FAM)-GCACTGGATACGACTCAGTT-(FQ)-3’ |
| **hsa-miR-205-5p**  **(miR-205)** | RT: 5’-GTCGTATCCAGTGCAGGGTCCGAGGTATTCGCACTGGATACGACCAGACT-3’  Forward: CCTCCTTCATTCCACCGGA  Probe: 5’-(FAM)-GCACTGGATACGACCAGACT-(FQ)-3’ |
| **hsa-miR-126-3p**  **(miR-126)** | RT: 5’-GTCGTATCCAGTGCAGGGTCCGAGGTATTCGCACTGGATACGACGCATTA-3’  Forward: 5’-CCGCTCGTACCGTGAGTAAT-3’  Probe: 5’-(FAM)-GCACTGGATACGACGCATTA-(FQ)-3’ |
| **hsa-miR-25-3p**  **(miR-25)** | RT: 5’-GTCGTATCCAGTGCAGGGTCCGAGGTATTCGCACTGGATACGACTCAGAC-3’  Forward: 5’-CCGCCATTGCACTTGTCTCG-3’  Probe: 5’-(FAM)-GCACTGGATACGACTCAGAC-(FQ)-3’ |
| **hsa-miR-125b-5p**  **(miR-125b)** | RT: 5’-GTCGTATCCAGTGCAGGGTCCGAGGTATTCGCACTGGATACGACTCACAA-3’  Forward: 5’-CGTCCCTGAGACCCTAACTT-3’  Probe: 5’-(FAM)-GCACTGGATACGACTCACAA-(FQ)-3’ |
| **hsa-miR-183-5p**  **(miR-183)** | RT: 5’- GTCGTATCCAGTGCAGGGTCCGAGGTATTCGCACTGGATACGACAGTGAA -3’  Forward: 5’- CGCTATGGCACTGGTAGAA -3’  Probe: 5’-(FAM)- GCACTGGATACGACAGTGAA -(FQ)-3’ |
