# Supplementary material for: Plasma miR-19b and miR-183 as Potential Biomarkers of Lung Cancer
Source: PLoS One. 2016 Oct 21;11(10):e0165261. doi: 10.1371/journal.pone.0165261 (PMC5074500; doi:10.1371/journal.pone.0165261)
Supplement: S2 Table — (DOCX) [file pone.0165261.s005.docx]

**Table S2. Stability of miR-16 expression in the study population.**

|  | **N1** | **N2** | **Median1+-SD1 (Lowest-Highest)** | **Median2+-SD2 (Lowest-Highest)** | **P (Mann-Whitney test)** |
| --- | --- | --- | --- | --- | --- |
| **HD vs LC** | 50 | 75 | 21.1+-2.56(16.4-24.7) | 20.8+-2.03(17.6-25.8) | 0.2910 |
| **SCC vs AD** | 53 | 18 | 20.85+-2.00(17.7-25.5) | 20.78+-2.08(19.1-24.9) | 0.8775 |
