# Supplementary material for: Plasma miR-19b and miR-183 as Potential Biomarkers of Lung Cancer
Source: PLoS One. 2016 Oct 21;11(10):e0165261. doi: 10.1371/journal.pone.0165261 (PMC5074500; doi:10.1371/journal.pone.0165261)
Supplement: S3 Table — (DOCX) [file pone.0165261.s006.docx]

**Table S3. Fold-change and significance levels of miRNA expression levels between groups.**

|  | **SCC vs HD** | | | **AD vs HD** | | | **LC vs HD** | | |
| --- | --- | --- | --- | --- | --- | --- | --- | --- | --- |
|  | **ddCq** | **Fold change**** | **P*** | **ddCq** | **Fold change** | **P** | **ddCq** | **Fold change** | **P** |
| **miR-21** | 0.36 | 1.29 | 0.2085 | 0.49 | 1.41 | 0.137 | 0.45 | 1.36 | 0.0441 |
| **miR-19b** | 1.45 | 2.72 | <0.0001 | 0.87 | 1.82 | 0.0018 | 1.25 | 2.38 | <0.0001 |
| **miR-126** | 0.97 | 1.95 | 0.0317 | 0.29 | 1.22 | 0.4936 | 0.50 | 1.41 | 0.1766 |
| **miR-25** | -1.49 | 2.80 | 0.0007 | -1.48 | 2.79 | 0.0839 | -1.09 | 2.13 | 0.0479 |
| **miR-205** | -2.23 | 4.69 | 0.0003 | 0.77 | 1.70 | 0.4224 | 0.07 | 1.05 | 0.9254 |
| **miR-183** | -1.42 | 2.67 | 0.1253 | -2.94 | 7.69 | 0.0368 | -2.49 | 5.61 | 0.0002 |
| **miR-125b** | 0.83 | 1.77 | 0.1021 | 0.11 | 1.08 | 0.8357 | 0.20 | 1.15 | 0.6617 |

*According to individual samples T-test or Welch t-test (where appropriate).

**Ratio of miRNA expression levels in plasma between two groups (2^-|ddCq|^)
